# Clinical Relevance of Cerebrospinal Fluid Antibody Titers in Anti-*N*-Methyl-d-Aspartate Receptor Encephalitis

**DOI:** 10.3390/brainsci12010004

**Published:** 2021-12-21

**Authors:** Meng-Ting Cai, Yang Zheng, Sa Wang, Qi-Lun Lai, Gao-Li Fang, Chun-Hong Shen, Yong-Feng Xu, Yin-Xi Zhang, Mei-Ping Ding

**Affiliations:** 1 Department of Neurology, Second Affiliated Hospital, School of Medicine, Zhejiang University, Hangzhou 310009, China; mengtingc93@zju.edu.cn (M.-T.C.); yangzh92@zju.edu.cn (Y.Z.); joysorrow2007@126.com (S.W.); janey_shen8808@163.com (C.-H.S.); xuyongfeng925@163.com (Y.-F.X.); zyx-neurology@zju.edu.cn (Y.-X.Z.); 2 Department of Neurology, Zhejiang Hospital, Hangzhou 310013, China; laiqilun@126.com; 3 Department of Neurology, Zhejiang Chinese Medicine and Western Medicine Integrated Hospital, Hangzhou 310003, China; morph2018@sina.com

**Keywords:** anti-*N*-methyl-d-aspartate receptor encephalitis, antibody titer, cerebrospinal fluid, psychiatric symptoms, modified Rankin scale

## Abstract

Anti-*N*-methyl-d-aspartate receptor (NMDAR) encephalitis is the most common autoimmune encephalitis. To date, there has been no study on the relationship between antibody (Ab) titers and clinical phenotype. This study aims to clarify the relationship between cerebrospinal fluid Ab titers and clinical manifestations of anti-NMDAR encephalitis at onset. Seventy-six consecutive patients with a definite diagnosis were enrolled. The relationship between Ab titers and different onset symptoms including psychiatric symptoms, seizures, and memory deficits were analyzed. We further investigated the correlation between Ab titers and clinical severity as assessed by the modified Rankin scale (mRS) and the clinical assessment scale for autoimmune encephalitis (CASE), respectively. The Ab titers had a median value of 1:10 (range 1:1–1:100). There was no significant difference in titers among various clinical factors including gender and combination of tumor and other diseases (each *p* > 0.05). Patients presenting with psychiatric symptoms at onset had higher titers than those with seizures (*p* = 0.008) and memory deficits (*p* = 0.003). The mRS scores revealed a significant but weak correlation with Ab titers (r = 0.243, *p* = 0.034), while CASE scores did not correlate with the titers (*p* = 0.125). Our findings indicated that the Ab titers were associated with the type of onset symptoms, with a higher level of patients with psychiatric symptoms. Regarding the clinical severity, the titers showed a weak correlation with the mRS, but no correlation with the CASE.

## 1. Introduction

Anti-*N*-methyl-d-aspartate receptor (NMDAR) encephalitis is the most common subtype of autoimmune encephalitis (AE), characterized by a prodromal phase and the subsequent progression of multiple symptoms, involving psychiatric symptoms, seizures, and memory deficits [1,2]. Immunoglobulin G (IgG) antibodies (Abs) to the NR1 subunit of NMDA-type glutamate receptors play an important role in this pathological process [3]. The detection of these Abs in serum and/or cerebrospinal fluid (CSF) is essential for a definite diagnosis. CSF IgG testing has shown a better performance than that of the serum, with a higher sensitivity and specificity [4]. Moreover, higher Ab titers have been associated with a more severe functional disability, with a modified Rankin scale (mRS) score of over 2 [4]. The increase of CSF titers also has a better correlation with clinical relapses (new onset of other symptoms or an increase in the mRS score by over one point) [4]. To date, the relationship between Ab titers and the disease phenotype or activity remains unknown. Therefore, in a cohort of anti-NMDAR encephalitis patients in China, we aimed to investigate the clinical implications of CSF Ab titers in terms of disease phenotype and activity.

## 2. Methods

### 2.1. Patients

Our retrospective study included 76 consecutive patients from the Second Affiliated Hospital School of Medicine Zhejiang University between July 2014 and October 2020. Inclusion criteria were: (1) aged ≥ 16 years; (2) cell-based analyses (CBA) for CSF samples at the acute phase of disease before immunotherapy; (3) first attack and definite diagnosis of anti-NMDAR encephalitis using the criteria of Graus et al. [5]. Exclusion criteria were: (1) combination with other antibodies against neuronal and/or glial antigens; (2) complicated by central nervous system infections (e.g., neurosyphilis, viral encephalitis, demyelinating diseases and others); (3) suffering from other severe neurological or primary psychiatric complications (e.g., brain tumor, stroke, myasthenia gravis, and others). Our study was approved by the ethics committee of the Second Affiliated Hospital School of Medicine, Zhejiang University.

### 2.2. Data Collection

We defined the onset phenotypes according to their initial symptoms, including psychiatric symptoms, seizures, memory deficits, and others. A series of potential influencing factors on Ab titers were collected according to the methods of previous studies [6,7,8,9,10,11], including the age of onset, disease duration, gender, other autoimmune complications, prodromal symptoms (headache, fever, upper respiratory symptoms, vomiting, diarrhea, and other associated symptoms that developed before the onset of encephalitic symptoms) [12]. We also divided patients according to their age of onset into subgroups including young, middle-aged, and old patients (16–40, 41–60, and over 60 years, respectively). Another subgroup analysis was performed in patients with acute, subacute, or chronic onset (according to time from onset to disease climax: 0–14, 15–30, and over 30 days). The clinical severity was evaluated with both the mRS and the Clinical Assessment Scale for Autoimmune Encephalitis (CASE) by 2 independent neurologists who were blind to the medical records at admission. For discrepant cases, the evaluating neurologists had a discussion to reach a final consensus. In brief, the mRS was mainly for assessment of motor functions, rated on a scale of 0 to 5 [13]. The CASE was developed with a relatively comprehensive profile of AE, with 9 items adding up to a maximum total score of 27—including the most common symptoms of psychiatric symptoms, seizures, memory deficits, etc. [14].

### 2.3. Ab Test

The CSF samples were collected within 1 week after admission before immunotherapy. Anti-NMDAR Ab was detected by cell-based indirect immunofluorescence assay using a commercial kit (Euroimmun, Lübeck, Germany). Testing was performed with undiluted samples. Positive samples that were reported by 2 experienced readers based on independent interpretations [15] were endpoint titrated with the Fc Ab (1:1, 1:3.2, 1:10, 1:32, 1:100, 1:320 etc.). The Ab titer was determined based on the last specific signal of dilution by 2 other independent, experienced technicians.

### 2.4. Statistical Analyses

The results were described as number (percentage) and median (interquartile range [IQR]). The Kolmogorov–Smirnov test was performed to evaluate the distribution of the variables. Categorical data were analyzed by Chi-square test or Fisher’s exact test. Data with non-normal distributions were calculated by nonparametric tests, such as the Mann–Whitney U test for 2 groups, or the Kruskal–Wallis H test for 3 groups with Dunn’s post hoc, adjusted by Bonferroni’s method. Spearman correlation coefficients were calculated between Ab titers (converted to a negative logarithmic scale) and other continuous variables or rank variables, and were judged as having negligible correlation (r < 0.20), weak (0.20–0.39), moderate (0.40–0.69), strong (0.70–0.89), and very strong (0.90–1.00) correlations [16]. Statistical significance was set at *p* < 0.05. R (version 4.0.2) was used for statistical analyses.

## 3. Results

### 3.1. Characteristics of Patients

Of the 76 patients enrolled in our study, 53.9% were female. The median age at onset was 29.0 years (IQR = 22.0–43.0), and the median duration was 17.5 days (IQR = 7.0–30.0). A range of 16 patients had concomitant systematic autoimmune diseases, including 7 (9.2%) with thyroid diseases and 9 (11.8%) with other connective tissue diseases. Three (3.9%) patients had ovarian teratomas. A majority of 38 (50.0%) patients showed a series of prodromal symptoms. With regard to the onset symptoms, psychiatric symptoms (i.e., delusions, hallucinations, disinhibition, aggression), seizures, and memory deficits were the most common manifestations sequentially (47.4%, 28.9%, 17.1%, respectively). Yet, 5 (6.6%) patients presented with other symptoms (3 with headache, and 2 with weakness). Above all, 17 (22.4%) patients had a monosymptomatic onset, including 7 with psychiatric symptoms, 6 with seizures, and 4 with memory deficits. Disease severity was assessed by scores on the mRS and CASE, with a median score of 2 (IQR = 1–3) and 3 (IQR = 2–6) respectively. The titers of the CSF Ab ranged from 1:1 to 1:100, with a median level of 1:10. Table 1 displays an overview of the demographic and clinical characteristics of patients.

### 3.2. Relationship between CSF Ab Titers and Demographic Characteristics

**Figure 1:**
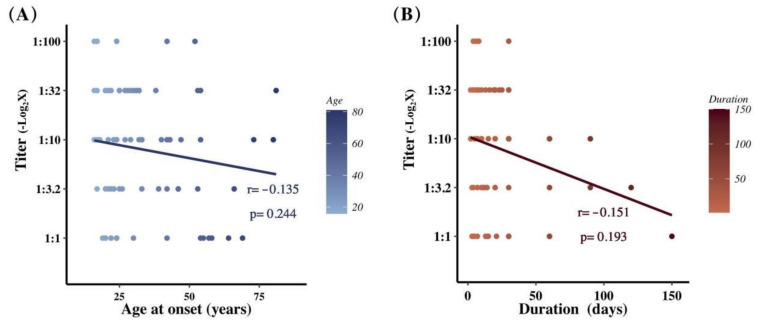
The distribution scatter plots of antibody titers by age of onset (**A**) and disease duration (**B**). The vertical axis shows the antibody titers after negative-logarithmic transformation, and the horizontal axis shows the time lapse.

The relationships between Ab titers and clinical factors were analyzed. As shown in Figure 1, with a negative-logarithmic transform (−Log_2_), the titers showed no statistically significant correlation with the age of onset or duration (*p* = 0.244, *p* = 0.193, respectively). Similarly, no significant difference in titers was found between the following dichotomous subgroups: those divided by gender (*p* = 0.081), by the combination of autoimmune diseases (*p* = 0.618), by tumors (*p* = 0.387) and by prodromal symptoms (*p* = 0.835).

### 3.3. Relationship between CSF Antibody Titers and Clinical Phenotypes

**Figure 2:**
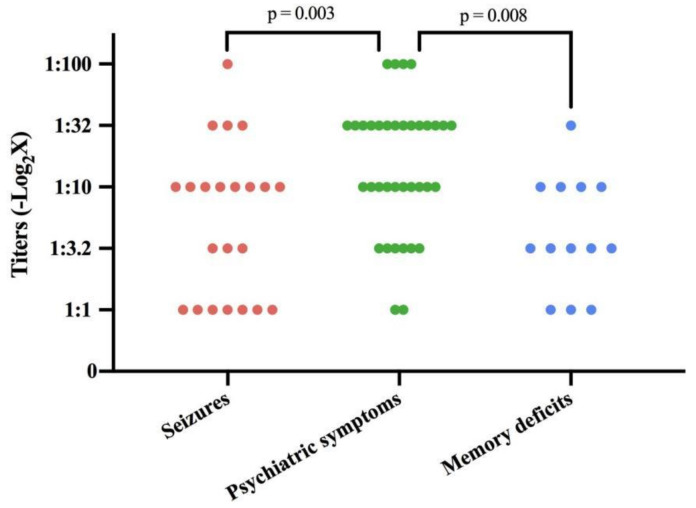
Cerebrospinal fluid antibody titers in patients with different onset symptoms.

The relationship between the Ab titers and onset phenotypes was analyzed (Table 2 and Figure 2). Among the three most common presentations, including seizures, psychiatric symptoms and memory deficits, patients with psychiatric symptoms had a significantly higher Ab titer (*p* < 0.001). Post-hoc analysis showed significantly higher titers in the psychiatric symptoms group when compared with patients with seizures (*p* = 0.008) and memory deficits (*p* = 0.003), respectively.

**Figure 3:**
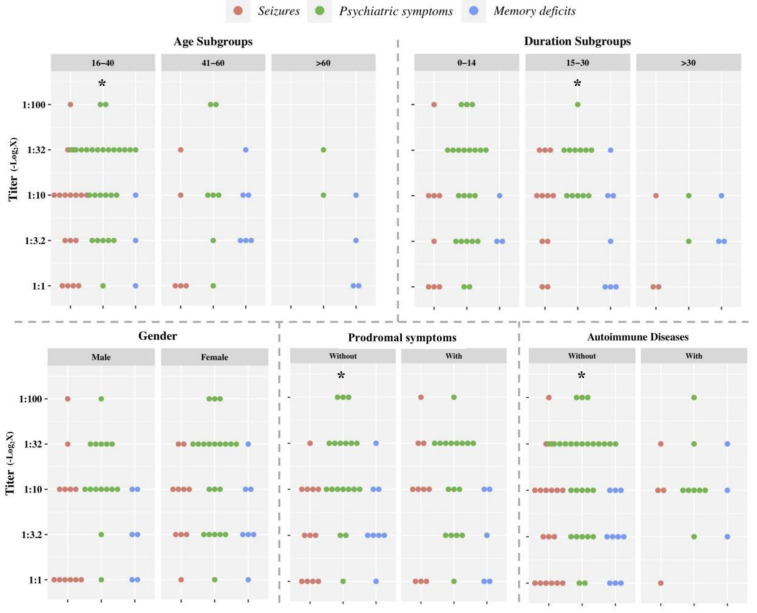
Cerebrospinal fluid antibody titers (with negative-logarithmic transform) compared between 3 onset-phenotype subgroups according to each factor (* with a significant difference between the 3 subgroups).

We then explored the confounding factors that might affect the performance of titers between these three phenotypes. In general, only two patients with ovarian teratomas showed a titer of 1:32 and 1:3.2, respectively. Further comparisons indicated that, within subgroups of younger age (16–40 years) and subacute phases (15–30 days), significantly higher titers were observed in patients with psychiatric symptoms than those presenting with the other two symptoms (Figure 3, detailed in Table 2). Additionally, in males, higher titers were observed in patients with psychiatric symptoms than in those with seizures (*p* = 0.032). Yet, there was no significant difference among patients with different autoimmune diseases or prodromal symptoms.

### 3.4. Relationship between CSF Antibody Titers and Disease Severity

**Figure 4:**
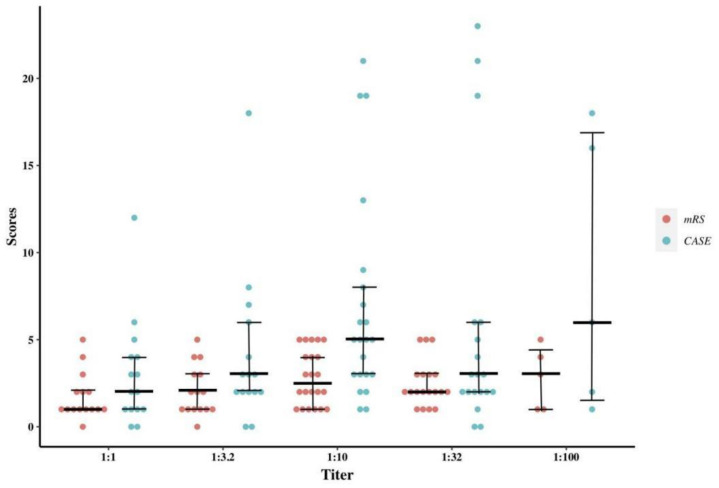
Relationship between mRS and CASE scores and antibody titers. The horizontal thick black lines indicate the median scores of each group, and the upper and lower lines represent the first and third quartiles. Abbreviations: CASE, clinical assessment scale for autoimmune encephalitis; mRS, modified Rankin scale.

The relationship between the Ab titers and the scores of the mRS and CASE was exhibited in Figure 4. Subgroup analysis was further performed in patients with different onset symptoms, genders, and specific complications (Table 3). To note here, we grouped items of the CASE corresponding to the 3 onset symptoms (seizures, psychiatric symptoms and memory deficits) when analyzing their correlation with Ab titers. The Ab titers of the memory deficits subgroup had a moderate correlation with the scores of the corresponding item in CASE (r = 0.608, *p* = 0.027). Both the scores of the mRS and CASE item corresponding to psychiatric symptoms revealed a significant but weak correlation with Ab titers (r = 0.243, *p* = 0.034; r = 0.316, *p* = 0.005, respectively). In the subgroup analysis, females revealed a significant yet weak correlation with scores for psychiatric symptoms (r = 0.332, *p* = 0.034).

## 4. Discussion

Our study found that patients with psychiatric symptoms had significantly higher Ab titers than those with seizures and memory deficits. Furthermore, patients starting at a young age and those with a subacute phase had higher titers as well. The Ab titers also revealed a weak correlation with clinical severity, as scored by mRS.

Our finding that patients with psychiatric symptoms had higher CSF Ab titers was partially consistent with previous findings in serum [17,18,19]. One previous study showed that patients with higher Ab titers more often presented with psychiatric symptoms as the initial symptom, with a higher severity [17]. Another study found elevated serum Ab levels in first-episode patients with schizophrenia [19]. Anti-NMDAR Ab might lead to neurological symptoms via the dysregulation of neurotransmitters, impairment of synaptic plasticity [20] or excitatory/inhibitory imbalance [9,21]. In particular, the preferential targeting of anti-NMDAR Ab at the limbic system might explain the association between high Ab titers and psychiatric symptoms [22]. Furthermore, memory deficits and seizures were related to Ab-mediated damage to the hippocampus, which also required a high level of Abs [8,23,24]. We hence proposed that the phenotype was dependent on the degree of neuronal damage, which was positively correlated with the level of Ab titers. Accordingly, psychiatric symptoms might be associated with a more complicated neural network, which required more Abs to induce [9]. Notably, in our study, in patients aged 16–40 years with a disease duration of 15–30 days, significantly higher CSF Ab titers were observed in patients with psychiatric symptoms than in those with other initial symptoms. In addition, in male patients, those with psychiatric symptoms had higher titers than those with seizures as well. Future large-sample multi-center studies should attempt to confirm those findings.

Several studies have focused on the relationship between Ab titers and clinical severity, as reflected by ICU admission, ventilator use and the presence of tumors [25,26]. Yet, no study has concentrated on their correlation to severity scales such as the mRS and CASE. Consistent with experience from clinical practice, our study showed a weak correlation between Ab titers and the mRS score in the whole cohort. However, confounding factors such as the Ab titers and Ab affinity might interfere with the results [16,27]. The Ab affinity has previously been found to have a determinant role in disease severity, showing a positive correlation with pathogenicity [28]. Yet, the relationship between the titers and affinity remained unknown. It was also unclear whether all the produced Abs were involved in pathogenesis of the disease. Meanwhile, other substances in the CSF such as albumin or globulins might also affect the Ab affinity, and therefore, the outcome of disease presentation [4,29,30]. In our study, we found inconsistent results in the mRS and CASE when assessing their correlations with Ab titers. The mRS had a score range of 0–5 and mainly represented motor function in AE. In contrast, the CASE had a more comprehensive representation of symptoms, with a wide range of total scores of 0–27. The scattered distribution of the CASE scores might contribute to the lack of correlation with Ab titers [14,31].

However, in the whole cohort, no significant relationship was found between the CSF Ab titers and demographic characteristics including age at onset, disease duration and gender, as well as other factors including concomitant systemic autoimmune diseases, allergies, and tumors. However, earlier studies have suggested that patients with teratomas, protracted clinical courses or persistent symptoms were more likely to have higher Ab titers in the CSF [4,27]. In fact, in our cohort, only three patients had teratomas, making the results prone to bias. Compared with the higher rate of teratomas in the study by Titulaer et al. (36.6%) [32], our lower rate (3.9%) was close to another Chinese multi-center study (7.5%) [33]. This discrepancy might be associated with our limited sample size and selection bias (excluding patients diagnosed with only serum Abs). Meanwhile, regional, ethnic and sexual differences might be involved as well. Moreover, we did not test the titers dynamically. The relationship between titers and disease activity should be further investigated. The disease course was also associated with the onset patterns and the subjective decisions of caregivers, as patients with seizures were often admitted with a shorter disease course, while those with cognitive deficits had an insidious onset, slow progression and usually a longer disease course. Above all, individualized longitudinal tracking of Ab titers will be helpful for their clinical utilization. Meanwhile, the significant findings in some subgroups, i.e., the correlation between memory deficits and the female subgroup and the scores of different CASE items, were still limited here by an insufficient sample size, and require further validation in larger cohorts.

Several limitations existed in this study. Firstly, the sample size of this single center was relatively small. Secondly, the onset symptoms mainly depended on the caregivers, and so might have been susceptible to subjective bias. Thirdly, we should acknowledge that our cohort had a relatively low titer ratio compared with that previously reported in Europe and America. Our CBA, performed with a commercial kit, might be partially responsible, as it might be insensitive to some antigens and prone to indeterminate results [34,35]. Otherwise, it showed a rapid and specific performance in most cases and is widely used in clinical laboratories. Thus, further detection could be performed by rodent brain immunohistochemistry followed by research-based CBA. Moreover, even though the Abs were detected within 1 week after admission, interindividual differences were still present given inconsistent rate of disease progression. Above all, larger multicenter studies with multiple assays are needed to validate our results.

## 5. Conclusions

Our study made several noteworthy contributions to the CSF Ab titers of anti-NMDAR encephalitis. Patients starting with psychiatric symptoms showed a higher titer than those with seizures and memory deficits, especially among youths in the subacute phase. Yet, the level of the titers was unaffected by age, duration, gender or other complications within 15–30 days of onset. Regarding the clinical severity, the titers showed a weak correlation with the mRS, but no correlation with the CASE. Further larger prospective studies are needed to better validate our findings, and related experiments are urgently required to explore their potential mechanisms.

## Figures and Tables

**Table 1:** Demographic and clinical characteristics of anti-NMDAR encephalitis.

	Patients (*n* = 76)
Demographic data	
Male, *n* (%)	35 (46.1%)
Age at onset, years (median, IQR)	29.0 (22.0–43.0)
Disease duration, days (median, IQR)	17.5 (7.0–30.0)
Personal history, *n* (%)	
Autoimmune diseases	16 (21.1%)
Tumors	3 (3.9%)
Prodromal symptoms	38 (50.0%)
Phenotype at onset, *n* (%)	
Psychiatric symptoms	36 (47.4%)
Seizures	22 (28.9%)
Memory deficits	13 (17.1%)
Others	5 (6.6%)
Clinical severity (median, IQR)	
mRS	2 (1–3)
CASE scores	3 (2–6)
CSF features	
Titers (median, IQR)	1:10 (1:3.2–1:32)

CASE, clinical assessment scale for autoimmune encephalitis; CSF, cerebrospinal fluid; IQR, interquartile range; mRS, modified Rankin scale; NMDAR, *N*-methyl-D-aspartate receptor.

**Table 2:** Comparison of cerebrospinal fluid antibody titers among groups with different onset symptoms.

	Numbers of Patients, *n* (%)	Comparison among Three Groups	*p* Values of Post-Hoc Tests		
			Seizures versus Psychiatric Symptoms	Seizures versus Memory Deficits	Psychiatric Symptoms versus Memory Deficits
Total cohort	71 (100.0)	0.003	0.008	0.619	0.003
Onset age (years)					
16–40	47 (66.2)	0.022	0.023	0.415	0.046
41–60	18 (25.4)	0.362			
>60	6 (8.5)	0.095			
Disease duration (days)					
0–14	33 (46.5)	0.171			
15–30	30 (42.3)	0.016	0.046	0.348	0.007
>30	8 (11.3)	0.529			
Gender					
Male	33 (46.5)	0.029	0.032	0.626	0.260
Female	38 (53.5)	0.857			
Autoimmune diseases					
With	15 (21.1)	0.873			
Without	56 (78.9)	0.002	0.009	0.410	0.002
Prodromal symptoms					
With	32 (45.1)	0.119			
Without	39 (54.9)	0.007	0.004	0.760	0.028

Each row corresponds to a separate comparison group, and the second column shows the number of patients in that group. The *p*-values of the titer comparison between different onset-symptom subgroups are shown in columns 3–6 respectively. Kruskal–Wallis H test with Dunn’s post-hoc test is used for comparisons between 3 groups, and Mann–Whitney U test is used for comparisons between 2 groups.

**Table 3:** Correlation between cerebrospinal fluid antibody titers and severity according to mRS or CASE scores.

	mRS	CASE			
		Total Scores	Seizures	Psychiatric Symptoms	Memory Deficits
Total cohort	0.034 * (r = 0.243)	0.125	0.302	0.005 * (r = 0.316)	0.499
Subgroups of onset symptoms					
Seizures	0.731	0.548	0.452	0.72	0.325
Psychiatric symptoms	0.91	0.848	0.479	0.603	0.91
Memory deficits	0.362	0.079	0.627	0.515	0.027 * (r = 0.608)
Subgroups of genders					
Female	0.053	0.052	0.498	0.034 * (r = 0.332)	0.546
Male	0.203	0.510	0.406	0.089	0.644
Subgroups with complications					
Autoimmune diseases	0.993	0.788	0.211	0.384	0.952
Prodromal symptoms	0.191	0.381	0.065	0.175	0.948

Each row corresponds to a separate comparison group. The *p* values represent the results of the Spearman’s rank correlation coefficient for the titer, with the severity score (mRS or CASE) in the corresponding column. * The value of r is given when *p* < 0.05. Abbreviations: CASE, clinical assessment scale for autoimmune encephalitis; mRS, modified Rankin scale.

## Data Availability

Anonymized data not published within this article will be made available upon reasonable request from any qualified investigator within 5 years after publication.
